# Lessons from co-production of evidence and policy in Nigeria’s COVID-19 response

**DOI:** 10.1136/bmjgh-2020-004793

**Published:** 2021-03-19

**Authors:** Ibrahim Abubakar, Sarah L Dalglish, Chikwe A Ihekweazu, Omotayo Bolu, Sani H Aliyu

**Affiliations:** 1 Institute for Global Health, University College London, London, UK; 2 Nigeria Centre for Disease Control, Abuja, Federal Capital Territory, Nigeria; 3 U.S. Centers for Disease Control and Prevention, Nigeria Country Office, Abuja, Nigeria; 4 Nigerian Presidential Taskforce on COVID-19, Abuja, Nigeria

**Keywords:** COVID-19, health policy, mathematical modelling, control strategies, health services research

## Abstract

In February 2020, Nigeria faced a potentially catastrophic COVID-19 outbreak due to multiple introductions, high population density in urban slums, prevalence of other infectious diseases and poor health infrastructure. As in other countries, Nigerian policymakers had to make rapid and consequential decisions with limited understanding of transmission dynamics and the efficacy of available control measures. We present an account of the Nigerian COVID-19 response based on co-production of evidence between political decision-makers, health policymakers and academics from Nigerian and foreign institutions, an approach that allowed a multidisciplinary group to collaborate on issues arising in real time. Key aspects of the process were the central role of policymakers in determining priority areas and the coordination of multiple, sometime conflicting inputs from stakeholders to write briefing papers and inform effective national decision making. However, the co-production approach met with some challenges, including limited transparency, bureaucratic obstacles and an overly epidemiological focus on numbers of cases and deaths, arguably to the detriment of addressing social and economic effects of response measures. Larger systemic obstacles included a complex multitiered health system, fragmented decision-making structures and limited funding for implementation. Going forward, Nigeria should strengthen the integration of the national response within existing health decision bodies and implement strategies to mitigate the social and economic impact, particularly on the poorest Nigerians. The co-production of evidence examining the broader public health impact, with synthesis by multidisciplinary teams, is essential to meeting the social and public health challenges posed by the COVID-19 pandemic in Nigeria and other countries.

**Summary box**

- In Nigeria, policymakers used a co-production model linking political decision-makers, health policymakers and academics from diverse disciplines to maximise the speed, relevancy and impact of scientific data and evidence to respond to COVID-19.
- This model allowed a multidisciplinary group to collaborate on issues arising in real time, with demonstrated impact on national decision making and apparently limiting the virus’ spread.
- Challenges of the co-production model included limited transparency, bureaucratic obstacles and an overly epidemiological focus on direct impacts of the disease compared with the social and economic effects of response measures.
- Integration of epidemiological, social science and economic analyses by multidisciplinary teams, in concert with policymakers, provides a strong path to meeting the twinned social and public health challenges created by COVID-19.

National responses to the COVID-19 pandemic have varied considerably.^1^ In Nigeria as elsewhere, policymakers made consequential decisions in a rapidly evolving and imperfect informational environment. Nigeria was at risk for an early epidemic given its air links with Europe and China,^2^ which raised the likelihood of multiple introductions. Furthermore, Nigeria faced a potentially catastrophic outbreak due the confluence of its high population density in major cities, particularly in urban slums and often poorly ventilated offices, as well as the high prevalence of infectious diseases and poor health infrastructure, conditions that hold in a number of other sub-Saharan African countries.

This practice paper describes policy responses taken to mitigate the COVID-19 pandemic in real time, along with potential explanations for observed trends in Nigeria. We present a model for the co-production of evidence between political decision-makers, health policymakers and academics, which was conceived in order to maximise the speed, relevancy and impact of data and scientific evidence brought to bear on far-reaching policy decisions. We examine the benefits and limitations of the evidence generation and decision-making process, discuss potential consequences and draw lessons for the way forward in Nigeria and other countries in sub-Saharan Africa and beyond.

## Co-production of evidence and policy for the COVID-19 response

**Figure 1:**
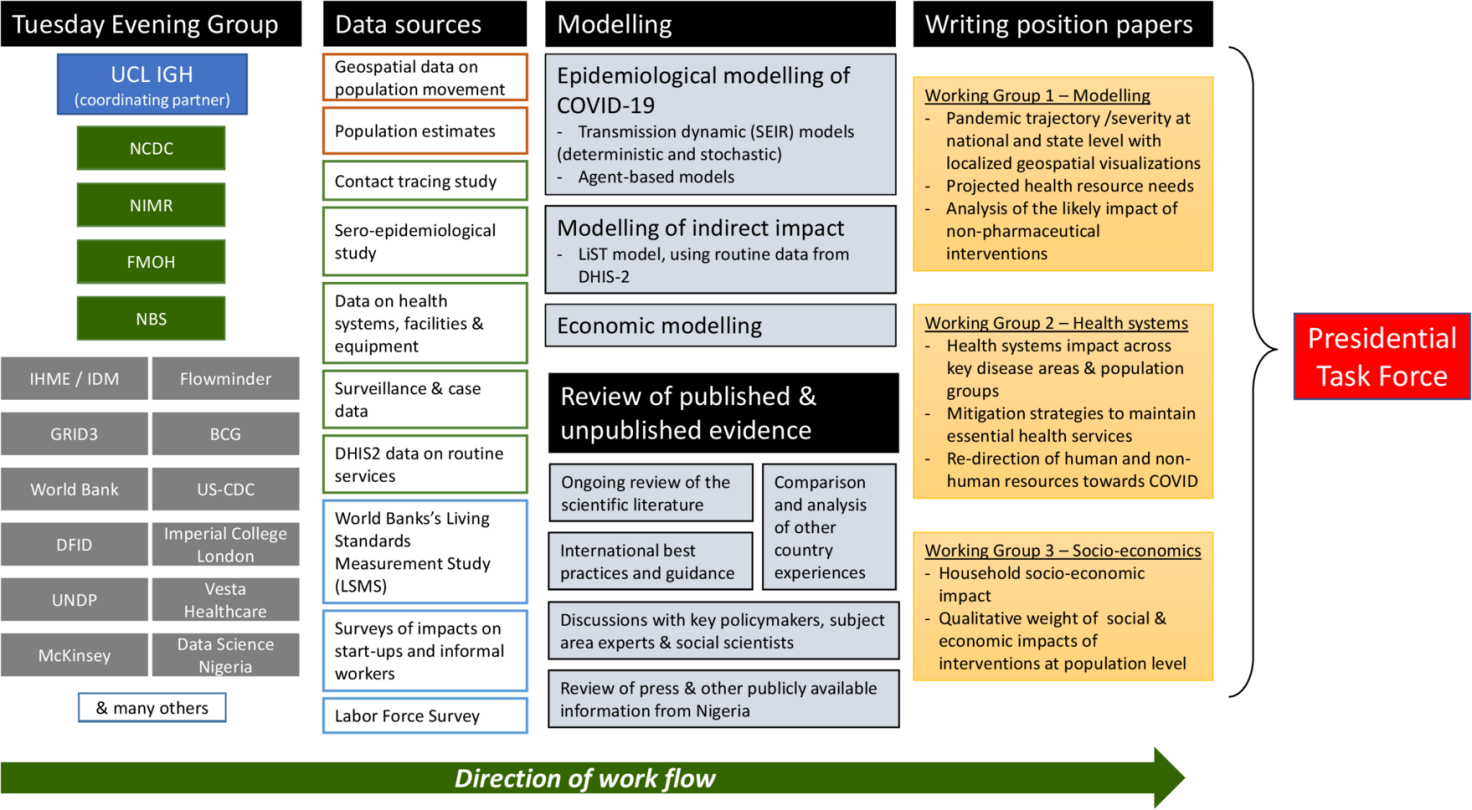
Membership and workflow of the PTF Advisory Group (Tuesday Evening Group).

On 26 January, Nigeria Centre for Disease Control (NCDC) established a national emergency response system with multiple workstreams and close liaison with state-level centres.^3^ The first case of COVID-19 in Nigeria was declared on 27 February 2020 in Ogun State.^4^ A Presidential Taskforce (PTF) on COVID-19 was inaugurated on 17 March to lead high-level policy decisions based on a Multisectoral Response Plan,^5^ with membership from a variety of ministries and agencies including health, finance, disaster management, aviation and foreign affairs. These policymakers initiated a process to gather, evaluate and synthesise evidence from the medical and social sciences by bringing together a group of stakeholders with academic, health policy and service expertise including epidemiologists, modellers, public health experts, social scientists and implementation partners. This group, called the PTF Advisory Group or ‘Tuesday Evening Group’ for its weekly virtual meetings, operated with the participation and leadership of government agencies including NCDC (which led the public health response), the National Bureau of Statistics (NBS), the National Institute for Medical Research and the Federal Ministry of Health, and collaborated to provide briefing papers responding to specific policy questions raised by the PTF (figure 1).

Between meetings, working groups met regularly to advance three work areas: epidemiological modelling and projections, health systems issues, and social and economic aspects of the response. A range of epidemiological and health systems data were provided from NCDC and other Nigerian government agencies; geospatial data and data on population mobility, food security and wages were also made available to the group. First, the modelling working group, used the data to parametrise a range of deterministic, agent-based and stochastic models to provide short-term projections on the course of the epidemic at national and state level (including expected incidence, illnesses requiring hospitalisation, and intensive care unit requirements and deaths), beginning when states hit the threshold of 100 cases. The models were used to compare different scenarios with respect to extending national and community lockdowns, testing and isolation strategies, imposing or lifting travel restrictions, opening borders and a variety of other non-pharmaceutical interventions (NPIs). Second, on a parallel track, the health systems working group studied the same questions from the perspective of the impact on health systems, with the goal of maintaining health services to the greatest extent possible. Finally, the socioeconomic working group looked at the wider societal impact of the disease, and particularly of response measures such as lockdowns and bans on mass gatherings, using survey data on how these measures affected living standards, small businesses and entrepreneurs, and learnings from other countries around the world.

Policy questions were fed from the PTF directly to the chair of the coordinating group via evening calls and communications. Policy questions guided the analyses of the three working groups, with different strands brought together and discussed during the Tuesday evening calls. Findings were combined into short, synthetic position papers limited to 1–2 pages, usually with much longer annexes to provide further detail as needed by policymakers.^6^ For example, the one-page briefing paper on post-lockdown interventions (provided to the PTF on 23 April) included 26 pages of annexes covering case studies of other countries’ interventions, a ranking of NPIs by feasibility and projected impact, a detailed description of quantitative modelling methodology and a discussion of considerations around the operationalisation of strategies to test, trace and isolate.

Position papers were provided first on a weekly or biweekly basis (often with accompanying PowerPoint slides) to feed into fast-moving decisions by the PTF. The PTF reported these outputs monthly to the president of Nigeria, through face-to-face meetings summarising position papers. The pace slowed as the epidemic was controlled and transmission rates began to wane in July and August. A total of some 20 position papers were provided, in addition to 25 state-specific papers describing epidemic projections and implications for the application of NPIs in each state. Position papers fed into major policy decisions taken by the Nigerian president, the PTF and other high-level decision-makers, including the extension of the cessation of movement (lockdown) order to more states (2 April) and in time (23 April); the gradual lifting of the lockdown order (4 May); orders on mandatory mask wearing, bans on interstate travel and mass gatherings, and curfews (4 May); precision lockdowns in states and specific local areas (18 May and 29 June); decisions to increase testing and create new laboratories (from 5 to 23 laboratories, as of 7 June)^7^; partial re-opening of schools (1 July) and the re-opening of international airports (4 September). A full description and linking of specific position papers with major policy decisions is provided in online supplemental table 1.

10.1136/bmjgh-2020-004793.supp1 Supplementary data

## Epidemic trajectory and possible endogenous explanations

Following the implementation of public health measures, case numbers declined but remained at a stable level (although they have since risen and declined again). Contrary to initial projections,^8^ the spread of COVID-19 infection and mortality in Nigeria has been fairly modest.^4^ Undertesting partly explains the low numbers, with only 864 104 tests done up to 17 December 2020, compared with tens of millions in many Asian and European countries, however, the proportion of positive samples remained relatively low during the phase with decreased detected cases, suggesting a true decline in incidence. Even after accounting for undertesting and the reported under-ascertainment of deaths,^9^ the impact of the epidemic has been considerably less than many estimates,^10^ although the situation continues to evolve. It should be noted that in Kano State (northern Nigeria), local questionnaire-based data^11^ reported more widespread COVID-19 specific symptoms, and a large number of deaths initially reported in the media,^12^ however, this ceased after an initial surge, and hospital beds were no longer full. Throughout the country, many temporary isolation/treatment centres have closed due to lack of patients. Unfortunately, following the relaxation of public health measures, and resumption of international travel, cases have increased resulting in new and widespread community transmission in December 2020 supporting the hypothesis that earlier measures contributed to the observed decline in June 2020. The level of infection in Nigeria was suggested by a large serological survey in October 2020, which found the prevalence of SARS-CoV-2 antibodies was 23% in Lagos and Enugu States, 19% in Nasarawa State, and 9% in Gombe State.^13^

A number of hypotheses have been advanced to explain the less-than-expected spread and severity of the pandemic in Nigeria and other sub-Saharan African countries. First, the much lower age structure of the continent’s population has surely played a role, given that COVID-19 mortality is considerably lower among young people.^14^ Given Nigeria’s median age of 17.8 years^15^ compared with Italy’s 44.3, China’s 37.4 and the USA’s 37.4 years, the majority of cases had milder forms of COVID-19. Another protective sociodemographic factor may be that elderly people in sub-Saharan are less likely to be concentrated in care homes, which have been ravaged by the virus in Western and high-income settings.^16^ Furthermore, outside major cities, sparsely populated rural areas may limit spread.^17^

There is emerging evidence that prior widespread exposure to other coronaviruses has led to cross immunity in Africa, as evidenced by high prevalence of serological cross-reactivity against SARS-CoV-2 in pre-COVID-19 pandemic plasma samples from sub-Sahara Africa.^18^ Other more exotic explanations have also been put forward, such as a Neanderthal-inherited gene, largely absent in Africans, which has been postulated to partly explain the observed lower mortality in Africa.^19^ A comprehensive accounting of the reasons for the more limited spread and mortality of SARS-CoV-2 in sub-Saharan Africa is still outstanding.

## Impact of the response and government intervention

Similar to other African countries, Nigeria likely benefited from the early initiation of public health interventions, guided by the advice and action of the PTF. The prompt action to institute movement restrictions and introduce NPIs, just a few weeks after the first confirmed case, potentially truncated the peak of the epidemic, thus allowing some immunity to develop in subsections of the population at a modest peak infection level.^20^ The first 100 days of the response were marked by a sociomedical response that included direct efforts to control the spread of the virus, and also frequent public communications via PTF press briefings, rumour surveillance and social media postings to keep the public informed.^7^ As a result, despite concerns that in poor communities, adherence to social distancing and hygiene guidelines would be low, surveys found some evidence of sustained high reported levels of hand washing and wearing of face coverings, even after lockdown had ended.^21^ However, it is unlikely that those in the poorest urban communities were able to adhere to effective social distancing. Nevertheless, Nigeria’s aggressive early response to COVID-19, leveraging existing preparedness and learning from countries where transmission began earlier, and coordinated by the high-level leadership of the PTF, likely slowed virus transmission.^3^

Many countries in sub-Saharan Africa have faced outbreaks in recent years, with lessons learnt, however, subsequent preparedness has been variable. At the end of 2019, Nigeria and almost all African countries had undertaken a Joint External Evaluation of the International Health Regulations, which showed gaps in several key technical areas needed for countries to address outbreaks in a coordinated manner, but also highlighted improving competencies in real-time surveillance and immunisation.^22^ A previous European-led modelling study of the preparedness of African countries and vulnerability to COVID-19 described Nigeria as having a moderate risk and high vulnerability.^8^ In Nigeria, before the country’s first reported case, NCDC introduced a COVID-19 module in its Surveillance, Outbreak Response Management and Analysis System, obtained PCR testing ability and set up the national response system with multiple workstreams.^3^

Importantly, although the spread and severity of the disease appears to have been limited by policy, these decisions had trade-offs, especially in terms of the socioeconomic well-being of the poorest segments of the population. Lockdowns, compounded by self-imposed distancing, the closing of markets and businesses and travel restrictions led to skyrocketing food and transport prices, hoarding and post-harvest food loss at both farm and market levels, resulting in a ‘hidden’ food security crisis among the country’s most vulnerable populations.^23^ Phone surveys conducted by NBS and the World Bank revealed widespread economic suffering. In April/May, a national survey (n=1950 households) found that 42% of respondents who were working before the outbreak said they were not currently working due to COVID-19, and 35%–59% reported difficulties affording staple foods.^21^

Palliative measures introduced by the Nigerian government to mitigate the socioeconomic impact of the pandemic and response measures, such as distribution of food items and conditional cash transfers, have been criticised as being inadequate,^24^ and their intended effect was attenuated by poor coordination and inadequate fiscal provisioning.^25^ Human rights abuses were observed, including violent enforcement of lockdowns targeting marginalised communities.^26^ Increased rates of gender-based violence and domestic violence were also reported during lockdowns in Nigeria,^27^ as in other countries around the world. Further, children were kept out of school for several months, causing potentially critical learning losses and increasing inequities between rich and poor as schools serving wealthy communities continued with online learning. The most severe effects likely fell on girls, if patterns observed in the West African Ebola outbreak hold in the current pandemic.^28^ Continued disruptions to routine and emergency healthcare may also have had significant impacts on access to immunisation, maternal and child healthcare,^29^ and surveillance for other infectious diseases as well as treatment for diseases such as malaria.^30^ Fortunately, most of these services have returned to normal functioning but the long term consequences require specific catch-up investments and overall increases in the budget for health.

## Lessons from the Nigerian co-production model

Nigeria’s COVID-19 evidence co-production model was in many ways beneficial to the response, as it resulted in responsive evidence gathering and synthesis, drawing from diverse expertise, with a demonstrable impact on policy decisions. Open daily communication between the PTF Advisory Group (the Tuesday Evening Group) and the PTF ensured that position papers were directly relevant to the most pressing policy questions, and that evidence and recommendations were delivered in time to inform decisions. As a result, the position papers and other support provided valuable inputs into consequential early decisions on lockdown and the combinations of NPIs likely to be most effective in limiting transmission. Subsequent position papers directly influenced the approach taken for more targeted policy questions, including optimal test, treat and isolation strategies; restrictions on inter-state movement; the contribution of asymptomatic cases to transmission; precision lockdown; the need for cash transfers to mitigate the impact on socioeconomic well-being; school closures; and international border re-opening. The group’s diverse expertise, organised into topical work areas, allowed for substantive input on this broad range of policy questions. Furthermore, the academic leadership of the group and primary accountability to the PTF limited the influence of any single donor or technical partner despite the utilisation of funding and resources from multiple organisations and governments. This structure ensured government action was not overwhelmed by the strategic agendas of powerful outside actors, as can occur in low-resource settings (Box 1).

Box 1 **Strengths, weaknesses, and outside limitations of the Nigerian evidence co-production model**

**Strengths**

- Establishment of a presidential taskforce bringing together key policy decisions-makers
- Timely provision of relevant information and analysis
- Multi-disciplinary team with diverse approaches, allowing for triangulation between different models and analysis tactics
- Linkage with Nigerian and non-Nigerian academics and experts
- Academic leadership of evidence co-production group limited influence of any single donor or financial partner

**Weaknesses**

- Limited ability for researchers to initiate position papers on topics they found relevant
- Lack of transparency in content of position papers and inputs into decision-making process
- Predominance of epidemiologists with relatively lesser participation of social scientists, economists, risk communication specialists, and other specialists
- Bureaucratic red-tape hindered important serological and contact tracing studies

**Outside limitations**

- Fractured governance, with lack of coordination between multiple tiers with responsibility for implementation
- Political dynamics and interests hampering response in some states
- Public distrust of public health system and in political leadership due to chronic lack of performance
- Economic impact of lockdown and COVID-19 resulting in fewer resources to spend on health
- Insecurity due to terrorism and other security risks hampered response in some parts of the country
- Health system weaknesses including limited testing capacity and ability to manage critically ill patients

This policymaking approach had some weaknesses (Panel). First, the directive nature of the PTF’s involvement meant that research teams were limited in their ability to initiate briefing topics, restricting the breadth of subjects for position papers and potential scope of useful advice. Second, the position papers initially had a limited circulation list. Although these papers have now been made widely available,^6^ the process limited external scrutiny, including by communities. Third, while the composition of the PTF Advisory Group (Tuesday Evening Group) was broad and included members of government, academic, civil society and the private sector, there was a predominance of epidemiologists and medical scientists in the group, with relatively fewer social scientists, communications experts and other specialists from diverse fields whose input is required for a comprehensive response to the pandemic. The immediate threat of a devastating epidemic meant that the initial focus of the PTF was the imperative to limit transmission, with the socioeconomic impact of response measures taking a secondary position. After the initial interventions were in place, attention was paid to social dimensions including food insecurity, the effect on informal labourers, increased domestic violence, and the negative impact of school closures on children and society.

Bureaucratic red tape was unfortunately a significant obstacle when it came to producing Nigeria-specific evidence needed to parametrise models and understand the course of the pandemic in country. Two planned studies, a serological survey and a contact tracing study, were severely hampered by slowdowns in funding and administrative arrangements caused by lockdowns and other challenges, where the relevant grants were being processed. While these two studies are currently underway, the data they will generate would have been much more useful in the early days of the response. Contractual processes between multiple organisations, while complex, can and must be expedited in the context of pandemics and other emergencies, and in the future all measures should be taken to ensure the relevant arrangements are in place in advance. On the Nigerian side, red tape was less of a barrier, as the creation of the PTF allowed policymakers to overcome ministerial divisions and make high-level decisions with relative ease.

The impact of the evidence co-production model was also constrained by limitations inherent to the Nigerian public health system, political system and economy. First, although the PTF led the national multisectoral response, Nigeria’s federal structure means that individual state governments led local implementation despite varying capacity. Decisions made at federal level were not always effectively translated into action for communities on the ground. In other instances, states were overly dependent on federal institutions. Other limitations were found in historical aspects of Nigeria’s political economy, such as its weak public health funding, particularly at primary healthcare level; a small and shrinking share of resources available for public health, including for the COVID-19 response; public distrust of government authorities and insecurity due to terrorism and other security risks.

## Conclusions

Contrary to long-standing narratives in global health, the COVID-19 response in many poor countries suggests that wealth is not the sole determinant of success.^31^ In its first phase, Nigeria’s response to the pandemic has been a qualified success, in that early action on lockdowns, border closures and adoption of a set of effective NPIs appears to have limited transmission; the response has also been used to strengthen surveillance and laboratory capacity. These decisions were largely made based on a flexible, rigorous, if imperfect, evidence co-production process. Nonetheless, the social and economic impact of COVID-19 has been harmful for many Nigerian families and individuals, with Nigeria’s efforts to address its significant wider health challenges compounded by the contraction of the economy.^32^ The government has launched a 12-month Economic Sustainability Plan^33^ to mitigate the economic impact of COVID-19. However, the national COVID-19 response should dedicate strong efforts towards minimising further health harms directly from SARS-CoV-2 infection and indirectly from interventions. The established laboratory and surveillance infrastructure should be utilised to support future public health action including vaccination and the management of future outbreaks. Integration of epidemiological, social science and economic analyses, by multidisciplinary teams and with the co-production of evidence with policymakers, will provide the best path forward to meeting the twinned social and public health challenges created by the COVID-19 pandemic in Nigeria and other countries.
