# Supplementary material for: Lessons from co-production of evidence and policy in Nigeria’s COVID-19 response
Source: BMJ Glob Health. 2021 Mar 19;6(3):e004793. doi: 10.1136/bmjgh-2020-004793 (PMC7985933; doi:10.1136/bmjgh-2020-004793)
Supplement: Supplementary data [file bmjgh-2020-004793supp001.pdf]

Supplementary Table. Position papers, analytical approaches and major policy decisions

Position Paper	Approaches used	Main Recommendation	Major Policy Decisions
**27th February to 30th May – Initial Actions**			
Lockdown	Modelling, evidence review	Approaches to inform cessation of movement	1st confirmed case (27th February)<br>PTF established (March 9th)<br>Initial limited travel ban (March 18th)<br>School closures (March 18th)<br>Lagos, FCT and Ogun lockdown (March 30th)
Population health and socio-economic well-being	Summary of bespoke surveys and literature	Informed decision socioeconomic support and precision lockdown	Food assistance and cash transfers (1st April)
Asymptomatic transmission	Literature review and modelling	Informed wider testing and isolation policy	Testing and duration of isolation (20th April)
Continuation of lockdown	Modelling, evidence review	2-week extension of lockdown	International travel ban extended (March 23rd)<br>Extension of lockdown to more states (April 2nd)<br>Extension of lockdown in time (April 13th)
**4th May to 30th May – Phase 1**			
Interventions post lockdown	Modelling, country experience	Choice of NPIs following relaxation of lockdown	Gradual easing of lockdown initiated (4th May)<br>Mandatory Mask wearing; mass gathering ban; ban interstate travel; curfews (May 4th)
Local strategy	Modelling	Informed precision lockdown plans	Precision lockdown by states (18th May)
Scaling up testing	Modelling, literature	Informed strategy for scaling up testing	Need to increase testing and new laboratories (May)
Africa epidemic size	Review of country experience	Informed discussion on whether Africa is different	No specific policy decision
**1st June to 27th July – Phase 2**			
Admission and discharge	Literature review	Informed decision on admission duration	Allowing self-isolation for non-severe cases (5th June)
State level analysis	Modelling, data analysis	Informed extension of local lockdown	Precision lockdown in specific local areas (29th June)
Interstate transmission	Modelling	Informed decision to allow domestic air travel	Lifting movement restriction between states (1st July)
Re-opening schools	Modelling, literature review	Re-opened schools should have infection mitigation measures	Partial re-opening (1st July)<br>States allowed to consider (September)
**30th July to 15th September – Phase 3**			
Re-opening borders	Modelling and other country experience	Informed discussion on partial lifting of the international travel ban	International airports reopened (4th September)
State level papers*	Modelling	Inform state discussions on more testing, palliatives and precision lockdown.	State level distribution of data (Multiple dates between June-Sept)
Timing of entry tests	Modelling, literature review	Inform discussion on pre-arrival, on entry and post arrival testing to lift self-isolation	Testing at day 7/8 after arrival (4th September)

* *Adamawa, Bauchi, Bayelsa, Borno, Delta, Ebonyi, Edo, Enugu, FCT, Gombe, Jigawa, Kaduna, Kano, Katsina, Kwara, Lagos, Nassarawa, Ogun, Ondo, Oyo, Plateau, Rivers, Sokoto, Yobe, Zamfara*
